# 

**Published:** 1897-01

**Authors:** 


### BOOKS RECEIVED.

The Diseases of the Male Urethra. By R. W. Stewart, M. D., M. R. C. S., Surgeon to Mercy Hospital, Pittsburg, Pa. One volume of 229 pages, post-octavo, illustrated by numerous wood-engravings. Muslin, $2.50 ; flexible morocco, $3.25. New York : William Wood & Company. 1896.

Remsen's Theoretical Chemistry. New (fifth) edition. Principles of Theoretical Chemistry, with special reference to the constitution of chemical compounds. By Ira Remsen, M. D., Ph. D., Professor of Chemistry in the Johns Hopkins University, Baltimore. New (fifth) and thoroughly revised edition. In one royal 12mo volume of 328 pages. Cloth, $2.00. Philadelphia and New York : Lea Brothers & Company, Publishers.

Diseases of the Stomach. A Text-Book for Practitioners and Students. By Max Einhorn, M. D., Instructor in Clinical Medicine at the New York Post-Graduate Medical School and Hospital ; Visiting Physician to the German Dispensary. Pp. 496. New York : William Wood & Company. 1896.

International Clinics. A Quarterly of Clinical Lectures on Medicine, Neurology, Surgery, Gynecology, Obstetrics, Ophthalmology, Laryngology, Pharyngology, Rhinology, Otology and Dermatology, and specially prepared articles on treatment. By professors and lecturers in the leading medical colleges of the United States, Germany, Austria, France, Great Britain and Canada. Edited by Judson Daland, M. D., (Univ. of Penna.) Philadelphia, Instructor in Clinical Medicine and Lecturer on Physical Diagnosis in the University of Pennsylvania ; Assistant Physician to the Hospital of the University of Pennsylvania, etc.; Fellow of the College of Physicians of Philadelphia. J. Mitchell Bruce, M. D., F. R. C. P., London, England, Physician to, and Lecturer on, the Principles and Practice of Medicine at the Charing Cross Hospital. David W. Finlay, M. D., F. R. C. P., Aberdeen, Scotland, Professor of Practice of Medicine in the University of Aberdeen ; Physician to, and Lecturer on, Clinical Medicine in the Aberdeen Royal Infirmary, etc. Volume III. Sixth series. 1896. Octavo, pp. xii.—344. Philadelphia : J. B. Lippincott Co. 1896.

Report of the Commissioner of Education for the Year 1894-95. Volume I. Containing Part I. Washington : Government Printing Office. 1896.

Annual Report of the State Board of Charities for the Year 1895. Transmitted to the legislature, March 25, 1896. Albany, N. Y.: Wynkoop Hallenbeck Crawford Company, State Printers. 1896.

Transactions of the Medical and Chirurgical Faculty of the State of Maryland, Ninety-eighth Annual Session, held at Baltimore, Md., April, 1896, also Semi-Annual Session, held at Belair, Md., November, 1895, and Special Memorial Meeting in Baltimore, December 14, 1895. Baltimore, Md.: The Deutsch Lithographing Company.

Transactions of the American Gynecological Society. Volume XXI. For the Year 1896. Philadelphia: Wm. J. Dornan, Printer. 1896.

Transactions of the Colorado State Medical Society, Twenty-sixth Annual Convention. By-Laws and List of Members. Denver, June, 1896. Published for the Society. Denver, Col. The Smith-Brooks Printing Company. 1896.

